# No Association of *BDNF*, *COMT*, *MAOA*, *SLC6A3*, and *SLC6A4* Genes and Depressive Symptoms in a Sample of Healthy Colombian Subjects

**DOI:** 10.1155/2015/145483

**Published:** 2015-10-08

**Authors:** Yeimy González-Giraldo, Andrés Camargo, Sandra López-León, Diego A. Forero

**Affiliations:** ^1^Laboratory of NeuroPsychiatric Genetics, Biomedical Sciences Research Group, School of Medicine, Universidad Antonio Nariño, Bogotá, Colombia; ^2^School of Nursing, Universidad de Ciencias Aplicadas y Ambientales (UDCA), Bogotá, Colombia; ^3^Novartis Pharmaceuticals Corporation, East Hanover, NJ 07936, USA

## Abstract

*Background*. Major depressive disorder (MDD) is the second cause of years lived with disability around the world. A large number of studies have been carried out to identify genetic risk factors for MDD and related endophenotypes, mainly in populations of European and Asian descent, with conflicting results. The main aim of the current study was to analyze the possible association of five candidate genes and depressive symptoms in a Colombian sample of healthy subjects. *Methods and Materials*. The Spanish adaptation of the Hospital Anxiety and Depression Scale (HADS) was applied to one hundred eighty-eight healthy Colombian subjects. Five functional polymorphisms were genotyped using PCR-based assays: *BDNF*-Val66Met (rs6265), *COMT*-Val158Met (rs4680), *SLC6A4*-HTTLPR (rs4795541), *MAOA*-uVNTR, and *SLC6A3*-VNTR (rs28363170). *Result*. We did not find significant associations with scores of depressive symptoms, derived from the HADS, for any of the five candidate genes (nominal *p* values >0.05). In addition, we did not find evidence of significant gene-gene interactions. *Conclusion*. This work is one of the first studies of candidate genes for depressive symptoms in a Latin American sample. Study of additional genetic and epigenetic variants, taking into account other pathophysiological theories, will help to identify novel candidates for MDD in populations around the world.

## 1. Introduction

Major depressive disorder (MDD) is a current challenge for mental health research [1]. Average global lifetime prevalence of MDD is around 11.2%, showing regional differences [2]. According to data from the Global Burden of Disease Study, MDD was responsible for 8.2% of global years lived with disability (YLDs) and for 2.5% of global disability adjusted life years (DALYs) [1], being the second cause of YLDs around the world [3] and the main neuropsychiatric disorder in terms of DALYs [4].

Heritability for MDD has been estimated about 0.37 [5, 6]. A large number of studies have been carried out to identify genetic risk factors for MDD [7, 8]. A field synopsis for MD genetics carried out meta-analyses for 20 polymorphisms in 18 genes and variants in 5 candidate genes were found to be significant (*APOE*,* GNB3*,* MTHFR*,* SLC6A3*, and* SLC6A4*) [9]. Recently, genome-wide association studies for MDD have been carried out, with no main significant results [7]. In the context of the study of endophenotypes for MDD [10, 11], several works have explored the possible association of candidate genes with depressive symptoms in healthy subjects [12–16]. Almost all of those studies included were carried out in populations of European and Asian descent [9, 17]. The main objective of this study was to test the possible association of five polymorphisms in candidate genes (*BDNF*,* COMT*,* MAOA*,* SLC6A3*, and* SLC6A4*) with depressive symptoms in a Colombian sample of healthy subjects.

## 2. Materials and Methods

### 2.1. Participants

One hundred eighty-eight healthy young subjects were included in this study, with a mean age of 22.2 ± 5.6 years: 137 females (72.9%) and 51 males (27.1%). Included subjects were unrelated, had all four grandparents born in Colombia, and were recruited from a university in Bogotá, Colombia. The population living in Bogotá, the capital city of Colombia, has a main Southern European genetic background, with historical admixture with Native Americans [18, 19]. Participants with self-reported history of neuropsychiatric diseases, such as depression or anxiety disorders, were excluded [20]. This study was approved by the respective Institutional Ethics Committee (Universidad Antonio Nariño) and all participants provided written informed consent [21].

### 2.2. Phenotypic Measurements

Participants completed a self-administered questionnaire, which was used to collect sociodemographic variables (age, sex, and personal and familial history of neuropsychiatric disorders). The Spanish adaptation [22, 23] of the Hospital Anxiety and Depression Scale (HADS) [24] was applied, which is a well-validated and commonly used scale to measure depressive symptoms [25, 26]. It includes 14 Likert-type items rated on a 4-point scale (0–3), total scores ranged from 0 to 42, and it has two subfactors: depression (HADS-D) and anxiety (HADS-A) [24], each one with 7 items (scores for HADS-D and HADS-A: 0–21 for each subfactor).

### 2.3. DNA Genotyping

Five functional polymorphisms in candidate genes were studied:* BDNF*-Val66Met (rs6265),* COMT*-Val158Met (rs4680),* SLC6A4*-HTTLPR (rs4795541),* MAOA*-uVNTR, and* SLC6A3*-VNTR (rs28363170). A salting-out method was used to extract genomic DNA from peripheral blood of participants. DNA samples were adjusted to a final concentration of 10 ng/*µ*L and stored at 4°C until being used [27]. Genotyping assays for the 5 candidate genes were carried out as previously described [18, 20, 28, 29], using methods based on qPCR and conventional PCR and agarose gel electrophoresis. PCR reactions were carried out in a Labnet MultiGene thermal cycler (Labnet International Inc., Edison, NJ, USA) and their products were separated at 140 V in 2% agarose gel stained with SYBR Safe (Invitrogen, Carlsbad, CA, USA). Polymerase chain reactions contained 2 *μ*L of genomic DNA (~50 ng), 1.5 mM of MgCl_2_, 10x reaction buffer, 1.0 *µ*M of each primer, 1 mM of dNTPs, 1 M of Betaine, and 0.8 U of Taq polymerase (Bioline, London, United Kingdom) in a total volume of 20 *μ*L. Fragment sizes were determined by comparison to molecular weight markers (HyperLadder V, Bioline, London, United Kingdom). A CFX96 Touch Real-Time PCR system (BioRad, Hercules, CA, USA) was used for genotyping methods based on qPCR. A random subsample (10% of subjects) was reanalyzed for both polymorphisms to assure consistency in the genetic results. Additionally, all genotypes were checked by two different researchers in order to confirm and validate the results [30].

### 2.4. Statistical Analysis

Identification of subjects with HADS values located ±3SD from the mean of the sample was used to detect extreme outliers. SNPStats program [31] was used for calculation of allele and genotype frequencies and for Hardy-Weinberg equilibrium analysis (with *χ* ^2^ test). It was also used for the exploration of the association of genotypes in candidate genes with quantitative measures of depressive symptoms (HADS-D scores), using a linear regression model, corrected for age and sex. Categorical analysis was based on the use of a common cutoff score (8) for HADS-D [32]. *χ* ^2^ test was used to compare genotype frequencies between the two groups (normal and high HADS-D scores). Exploration of gene-gene interactions was carried out with the Quantitative Multifactor Dimensionality Reduction (QMDR) approach (using the MDR software, version 3.0.2) [33, 34] and the Multilocus Genetic Profile (MGP) method [35]. A nominal *p* value of <0.05 was taken as statistically significant. Bonferroni method was used to correct for multiple testing: with 2 subscales and 5 polymorphisms, a *p* value <0.005 (0.05 divided by 10) was considered as a threshold for corrected *p* values.

## 3. Results

Genotype frequencies for the 5 candidate polymorphisms in our current study are shown in Table 1 and they were in Hardy-Weinberg equilibrium (*p* values >0.05, *χ* ^2^ test). Mean scores for HADS-D and HADS-A in the total sample were 3.44 (SE: 0.20) and 7.05 (SE: 0.27), respectively. No extreme outliers for HADS scores were identified in the current sample. Cronbach's alphas for HADS-D and HADS-A were 0.689 and 0.799, respectively.

A linear regression model, correcting for sex and age, did not show significant associations for any of the five candidate polymorphisms and HADS-D scores (nominal *p* values >0.05) (Table 1). A categorical analysis, comparing genotype frequencies between normal and high HADS-D scores, did not show significant differences (nominal *p* values >0.05) (Table 1). Results from the association analysis for the 5 candidate genes and HADS-A were also not significant (nominal *p* values >0.05). Exploration of possible gene-gene interactions did not find significant associations, using the QMDR method (*p* value: 0.25) or the MGP approach (*p* value: 0.858).

## 4. Discussion

MDD is a current challenge, around the world, for mental health research [1]. A large number of studies have been carried out to identify genetic risk factors for MDD [7], mainly in populations of European and Asian descent, with conflicting results [9]. Study of endophenotypes for MDD [10], which involves the exploration of the possible associations of candidate genes with depressive symptoms in healthy subjects [12–16], represents interesting approaches for the identification of novel candidates for MDD [11].

In the current study, we did not find significant associations for any of the five candidate polymorphisms and HADS-D scores (nominal *p* values >0.05) in a Colombian sample of healthy subjects. A categorical analysis, comparing genotype frequencies between normal and high HADS-D scores, defined with a commonly used cutoff score [32], did not show significant differences (nominal *p* values >0.05). Exploration of possible gene-gene interactions did not find significant associations, using the QMDR method or the MGP approach (*p* values >0.05).

MDD is main priority for health research in Colombia, as in other countries. Estimates of lifetime and 12 months' prevalence of MDD in Colombia are 11.8 and 5.3%, in comparison to other Latin American countries such as Mexico and Peru (7.6/3.7 and 6.4/3.7%, resp.) [2]. The HADS-D scale, a well-validated and commonly used tool [32], has been previously used for genetic studies of depression [36–39]. A search in HuGeNavigator [40] shows that 384 genes have been explored as candidates for MDD in 1180 studies, in comparison to 924/2259 for BP, which has lifetime prevalence of 1% [41].* BDNF* and* SLC6A4* genes are the two top candidate genes for MDD, in terms of number of published studies.

To our knowledge, this is one of the first genetic studies of depressive symptomatology in Latin American subjects. It highlights the importance of studying genetic factors for MDD in other ethnicities, particularly developing countries [42, 43]. Future studies with larger sample sizes using other validated depression scales (such as CES-D) and including psychiatric patients [10] will facilitate the identification of candidate genes for endophenotypes for depression in Latin American populations. The forthcoming application of exome sequencing approaches [44] will allow the discovery of rare variants for MDD, in conjunction with epigenomic approaches [45] and the study of common variants in genes involved in other pathophysiological mechanisms for MDD [46, 47].

## Figures and Tables

**Table 1:** Associations between depressive symptoms and *BDNF, COMT, SLC6A3, MAOA*, and *SLC6A4* genes in a Colombian sample.

Gene/SNP	Genotypes	Frequencies *n* (%)	HADS-D mean (SD)	HWE	*p* value^a^	*p* value^b^
BDNF-rs6265	G/G	145 (0.77)	3.46 (0.23)	0.094	0.98	0.88
	G/A	37 (0.19)	3.35 (0.44)			
	A/A	6 (0.03)	3.5 (1.12)			

COMT-rs4680	G/G	75 (0.40)	3.55 (0.32)	0.35	0.83	0.96
	G/A	92 (0.49)	3.40 (0.27)			
	A/A	21 (0.11)	3.19 (0.62)			

SLC6A3-rs28363170	10/10	117 (0.66)	3.6 (0.26)			0.34
	10/9	53 (0.30)	3.06 (0.34)	0.25	0.43	
	9/9	10 (0.06)	3 (0.68)			

MAOA-VNTR	4/4	77 (0.43)	3.42 (0.32)			0.11
	4/3	65 (0.36)	3.09 (0.28)	0.58^*∗*^	0.47	
	3/3	38 (0.21)	3.63 (0.49)			

SLC6A4-rs4795541	S/S	49 (0.26)	3.12 (0.4)			0.85
	S/L	92 (0.49)	3.68 (0.28)	0.88	0.47	
	L/L	45 (0.24)	3.43 (0.42)			

^*∗*^HWE calculated in females. ^a^Quantitative association with total scores for HADS-D. ^b^Categorical association with a cutoff point of 8 (normal and high HADS-D scores).

HWE: Hardy-Weinberg equilibrium (*p* value); BDNF: brain-derived neurotrophic factor; COMT: catechol-O-methyltransferase; SLC6A3: dopamine transporter; MAOA: monoamine oxidase A; SLC6A4: serotonin transporter.
